# High-Pressure Carbonation of Phosphogypsum for Calcium Carbonate Preparation and Crystal Modification Regulation

**DOI:** 10.3390/ma19132787

**Published:** 2026-07-01

**Authors:** Shiyu Huang, Dongmei Liu, Xiaoxiang Zhang, Taotao Zhang

**Affiliations:** 1 College of Civil Engineering and Architecture, China Three Gorges University, Yichang 443002, China; 2 Hubei Key Laboratory of Disaster Prevention and Mitigation, Yichang 443002, China; 3 China Nuclear Industry Huaxing Construction Co., Nanjing 210019, China; 18306782903@163.com

**Keywords:** phosphogypsum, high-pressure carbonation, NH_4_Cl leaching, organic additives, calcium carbonate

## Abstract

Phosphogypsum (PG) was used as a calcium source for preparing calcium carbonate (CaCO_3_) through NH_4_Cl leaching followed by high-pressure carbonation. The effects of NH_4_Cl concentration, liquid-to-solid mass ratio, temperature, and leaching time on Ca^2+^ extraction were investigated, and the effects of CO_2_ pressure, carbonation time, and NH_3_·H_2_O dosage on Ca^2+^ conversion were evaluated. The optimal conditions were an NH_4_Cl concentration of 1.5 mol/L, a liquid-to-solid mass ratio of 60:1, a leaching temperature of 25 °C, a leaching time of 60 min, a CO_2_ pressure of 1 MPa, a carbonation time of 10 min, and 12 vol% NH_3_·H_2_O addition. Under these conditions, the Ca^2+^ leaching rate and conversion rate reached 81.25% and 97.36%, respectively. The product obtained without organic additives was mainly spherical vaterite with partial particle agglomeration. Based on the optimized process, aspartic acid, glutamic acid, ethanol, and glycerol were introduced to regulate CaCO_3_ crystallization. Appropriate additive dosages further improved Ca^2+^ conversion, promoted calcite as the dominant polymorph, and produced well-dispersed spherical CaCO_3_ particles. Among the tested additives, glutamic acid and glycerol showed the strongest effects on crystal morphology regulation.

## 1. Introduction

Phosphogypsum (PG), a solid waste by-product generated by the wet-process phosphoric acid industry, is produced in large quantities annually in Yichang, Province, China, a major phosphate-resource region in the Yangtze River Basin [1,2]. In the context of China’s “Dual Carbon” goals, namely carbon peaking and carbon neutrality, the conversion of PG into calcium carbonate (CaCO_3_) through carbonation offers a promising strategy for both CO_2_ utilization and solid-waste valorization [3,4]. However, the practical utilization of PG for CaCO_3_ preparation depends largely on developing a simple, low-cost, and efficient route for extracting calcium from the gypsum phase.

CaCO_3_ exists in three major polymorphs, among which calcite is the thermodynamically stable phase under ambient conditions [5,6]. Carbonation processes are generally classified into direct and indirect routes. Direct carbonation is suitable for calcium-rich systems containing a high content of free calcium oxide (f-CaO). Because calcium in PG mainly exists as calcium sulfate, an indirect carbonation route was adopted in this study to facilitate calcium extraction and subsequent control of CaCO_3_ precipitation. It should be noted that direct carbonation of calcium sulfate has also been reported to be feasible, even at room temperature [7]. Previous studies [8,9,10,11] have commonly employed strong acid or alkali leaching for calcium recovery; however, these approaches may cause severe equipment corrosion and co-dissolution of impurities. In comparison, NH_4_Cl is a low-cost and widely available salt leaching agent. During NH_4_Cl leaching, CaSO_4_·2H_2_O, the dominant gypsum phase in PG, partially dissolves and releases Ca^2+^ into the leachate [12]. Qiao et al. and Chen et al. [13,14] independently used NH_4_Cl solutions to leach PG and subsequently prepared CaCO_3_ by bubbling CO_2_ into the Ca^2+^-rich leachate, achieving Ca^2+^ conversion rates of approximately 80%. The resulting products were predominantly vaterite, characterized by spherical particles and fine, loose CaCO_3_ aggregates on their surfaces, with noticeable agglomeration. After Ca^2+^ leaching in the indirect process, the subsequent carbonation stage using conventional CO_2_ bubbling is limited by the low solubility of CO_2_ and slow gas–liquid mass transfer, resulting in slow reaction kinetics, prolonged carbonation times, and relatively low Ca^2+^ conversion efficiencies. High-pressure carbonation can effectively alleviate these limitations. López-Periago et al. [15] carbonated a Ca(OH)_2_ suspension under ultrasonic stirring at 40 °C and 13 MPa for 10 min, achieving a Ca^2+^ conversion rate of 88%. The obtained CaCO_3_ was calcite with a rhombic morphology formed through polycrystalline aggregation.

Previous studies have mainly focused on optimizing leaching and carbonation conditions and have shown that CaCO_3_ polymorphs and morphologies can be regulated either by adjusting carbonation parameters or by introducing additives. Nevertheless, the regulation of CaCO_3_ polymorphs and morphologies in PG-derived calcium leachate systems, particularly under high-pressure carbonation conditions, remains insufficiently understood. Seo et al. [16] investigated the effect of ethanol concentration on CaCO_3_ formation by adding different amounts of ethanol to a Ca(OH)_2_ solution prior to CO_2_ bubbling. They found that increasing ethanol concentration gradually decreased the average particle size of the resulting CaCO_3_ and shifted the dominant polymorph from calcite to vaterite. Jin et al. [17] conducted carbonation in a Ca(OH)_2_–ethanol system at 40 °C, 10 MPa, and 600 rpm for 2 h. The products were mainly vaterite, appearing as porous microspheres with uniform morphologies and no obvious agglomeration. Konopacka-Łyskawa et al. [18] performed CO_2_ bubbling carbonation in a Ca(OH)_2_ system containing 0–20% ethanol or glycerol at 20 °C and 900 rpm. Pure calcite was obtained under all tested conditions. Without additives, the CaCO_3_ particles had an average size of 9 µm. As the organic solvent concentration increased, the solution viscosity increased, leading to a gradual reduction in particle size; in particular, the 20% glycerol system produced particles with a size distribution of 0.1–0.59 µm. Luo et al. [19] introduced aspartic acid solutions at different concentrations into Ca(OH)_2_ suspensions and prepared CaCO_3_ by CO_2_ carbonation, obtaining products dominated by vaterite. Yang et al. [20] prepared CaCO_3_ by mixing Ca(OH)_2_ powder with 0–10% aspartic acid or glutamic acid solutions, followed by CO_2_ aeration at 20 °C for 2 h. In the absence of amino acids, pure calcite with a particle size of 1–2 µm was obtained. Aspartic acid and glutamic acid can selectively adsorb onto specific crystal faces of thermodynamically stable calcite [20,21], thereby inhibiting its preferential growth. As a result, the dominant polymorph shifted to vaterite after amino acid addition, and the particle size decreased. These studies indicate that additives can significantly affect CaCO_3_ crystallization. Alcohols can regulate solution viscosity, stabilize the calcite phase, and control particle size [15,16,17]. In contrast, amino acids can coordinate with Ca^2+^ through their carboxyl groups during the prenucleation stage, thereby affecting the formation and stability of calcium-containing precursor species. In addition, their selective adsorption on specific calcite facets can inhibit the preferential growth of calcite, which may favor the retention of metastable vaterite. However, most of these conclusions were derived from atmospheric-pressure bubbling carbonation experiments using Ca(OH)_2_ as the calcium source [18,19]. This system differs substantially from the PG-derived calcium leachate system under high-pressure carbonation used in the present study. Therefore, the corresponding polymorph and morphology regulation mechanisms require further clarification.

NH_4_Cl was selected as the leaching agent because of its low cost, wide availability, and mild leaching conditions. During NH_4_Cl leaching, CaSO_4_·2H_2_O in PG partially dissolves, releasing Ca^2+^ and SO_4_^2−^ into the NH_4_Cl-containing leachate. The filter cake mainly consists of undissolved PG residues and less soluble impurity phases, while minor amounts of soluble impurity species may also enter the leachate. Therefore, the main objective of this study was to develop a simple and cost-effective route for extracting Ca^2+^ from PG and converting it into CaCO_3_ through high-pressure carbonation. First, the effects of the liquid-to-solid mass ratio, NH_4_Cl concentration, reaction temperature, and leaching time on the Ca^2+^ leaching rate were investigated. Subsequently, the effects of CO_2_ pressure, carbonation time, and NH_3_·H_2_O addition on the Ca^2+^ conversion rate were examined to optimize the carbonation process. Finally, aspartic acid, glutamic acid, ethanol, and glycerol were introduced to evaluate their effects on the Ca^2+^ conversion rate, CaCO_3_ polymorphs, and microscopic morphologies.

## 2. Materials and Methods

### 2.1. Materials

PG samples were collected from a phosphogypsum stockpile in Yichang, Hubei Province, China, and appeared as a grayish-brown solid. Before use, the samples were dried at 100 °C to constant mass, pulverized using a ball mill, and passed through a 0.15 mm sieve to obtain the pretreated feedstock.

**Figure 1:**
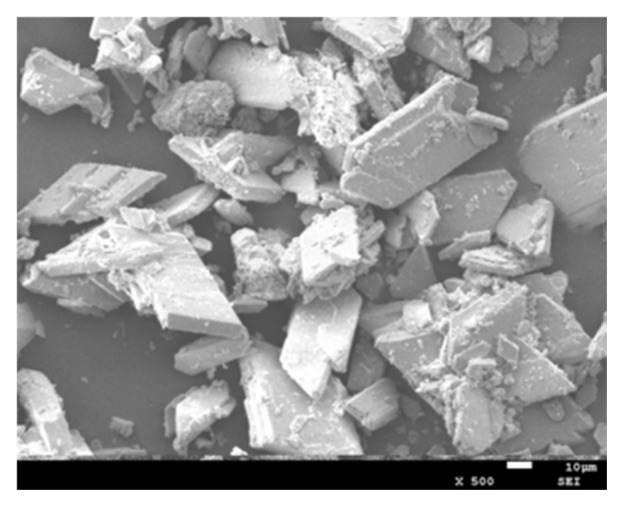
SEM image of raw PG.

**Figure 2:**
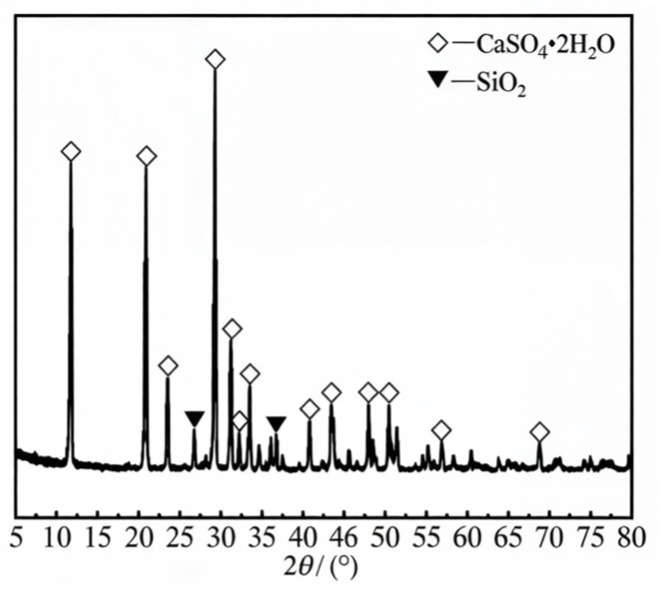
XRD pattern of raw PG. Symbols: ◇, CaSO_4_·2H_2_O; ▼, SiO_2_.

The chemical composition, microstructure, and X-ray diffraction (XRD) pattern of the raw PG are shown in Table 1, Figure 1, and Figure 2, respectively. All chemical reagents used in this study were of analytical grade unless otherwise stated. Ammonium chloride (NH_4_Cl, analytical grade, Sinopharm Chemical Reagent Co., Ltd., Shanghai, China), ammonia solution (NH_3_·H_2_O, concentration or purity, Sinopharm Chemical Reagent Co., Ltd., Shanghai, China), ethanol (C_2_H_5_OH, analytical grade, Sinopharm Chemical Reagent Co., Ltd., Shanghai, China), and glycerol (C_3_H_8_O_3_, analytical grade, Sinopharm Chemical Reagent Co., Ltd., Shanghai, China) were used. Aspartic acid (C_4_H_7_NO_4_, analytical grade, Shanghai Macklin Biochemical Co., Ltd., Shanghai, China) and glutamic acid (C_5_H_9_NO_4_, analytical grade, Shanghai Macklin Biochemical Co., Ltd., Shanghai, China) were also used.

### 2.2. Preparation of CaCO_3_

#### Leaching of Ca^2+^ from PG

Using a one-factor approach, 5 g of PG was added to NH_4_Cl solutions with different concentrations of 0.1–2.0 mol/L to prepare suspensions with liquid-to-solid mass ratios of 10:1–60:1. The suspensions were reacted at different temperatures of 25–85 °C for 10–120 min. After leaching, vacuum filtration was performed for solid–liquid separation. The filtrate was collected as the Ca^2+^-rich leachate, while the filter cake mainly consisted of undissolved PG residues and less soluble impurity phases.

Before carbonation, NH_3_·H_2_O solution was added to the Ca^2+^-rich leachate at different dosages of 4–16 vol%. The mixture was then transferred into a high-pressure reactor for carbonation. The carbonation reaction was conducted at 25 °C using CO_2_ with a purity of 99 vol% under pressures of 0.1–2.0 MPa for 4–15 min. After the reaction, the slurry was allowed to stand at room temperature for 2 h. The CaCO_3_ solids were then separated using a 0.1 µm membrane filter, thoroughly washed with deionized water, and dried at 80 °C to constant mass.

Aspartic acid, glutamic acid, ethanol, and glycerol were separately introduced into the Ca^2+^-rich leachate. The mixtures were carbonated for 10 min under the optimized conditions of 12 vol% NH_3_·H_2_O addition, 25 °C, and a CO_2_ pressure of 1.0 MPa. After carbonation, the slurry was filtered using a 0.1 µm membrane filter to collect the CaCO_3_ solids. The collected solids were thoroughly washed with deionized water and dried at 80 °C to constant mass.

### 2.3. Experimental Methods and Characterization

#### 2.3.1. Determination of Ca^2+^ Leaching Rate

The Ca^2+^ leaching rate reported in this study represents the experimentally measured apparent recovery of calcium from PG, rather than a theoretical equilibrium solubility value. First, 25 mL of the Ca^2+^-rich leachate was diluted tenfold with deionized water. Then, 25 mL of the diluted solution was collected, and the Ca^2+^ concentration was determined by EDTA titration. The amount of Ca^2+^ in the leachate, denoted as *n*_2_, was calculated from the volume of EDTA consumed. Finally, the Ca^2+^ leaching rate, *η*, was calculated using Equation (1):

(1) $$\eta =\frac{n_{2}}{n_{1}}\times 100\%$$

where *n*_1_ is the amount of Ca^2+^ in CaSO_4_·2H_2_O in PG (mol), and *n*_2_ is the amount of Ca^2+^ in the leaching solution (mol).

#### 2.3.2. Determination of Ca^2+^ Conversion Rate

After carbonation, the slurry was allowed to stand for 2 h and then filtered through a 0.05 µm membrane filter. The residual Ca^2+^ concentration in the filtrate was determined by EDTA titration, and the amount of residual Ca^2+^, denoted as n_3_, was calculated from the volume of EDTA consumed. The Ca^2+^ conversion rate, *α*, was then calculated using Equation (2):

(2) $$\alpha =\frac{n_{2}-n_{3}}{n_{2}}\times 100\%$$

where *n*_2_ is the amount of Ca^2+^ in the leaching solution (mol), and *n*_3_ is the amount of Ca^2+^ in the filtrate after carbonation (mol).

#### 2.3.3. Characterization Methods

The composition of PG and CaCO_3_ was qualitatively identified by X-Ray diffraction (XRD) using a SmartLab (9 kW) diffractometer (Rigaku Corporation, Tokyo, Japan). The morphology of PG and CaCO_3_ was observed using a 3200A scanning electron microscope (SEM) (Gwqnt Quantum, Hefei, China) operated at an accelerating voltage of 0.2–30 kV.

## 3. Results and Discussion

### 3.1. Process for CaCO_3_ Preparation via High-Pressure Carbonation of Phosphogypsum

#### 3.1.1. Leaching Rate of Ca^2+^ in PG

**Figure 3:**
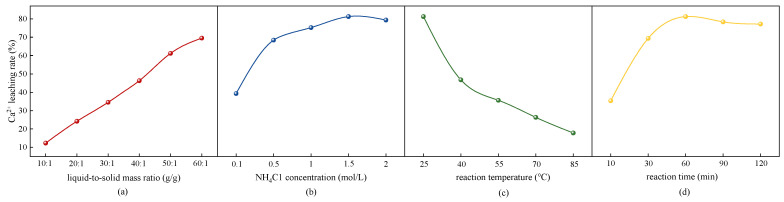
Effects of (**a**) liquid-to-solid mass ratio, (**b**) NH_4_Cl concentration, (**c**) reaction temperature, and (**d**) reaction time on the Ca^2+^ leaching rate.

Figure 3 shows the effects of the liquid-to-solid mass ratio, NH_4_Cl concentration, reaction temperature, and reaction time on the Ca^2+^ leaching rate.

After reaction with 0.5 mol/L NH_4_Cl at 25 °C for 60 min, the Ca^2+^ leaching rate increased markedly with increasing liquid-to-solid mass ratio. The maximum leaching rate of 69.5% was obtained at a liquid-to-solid mass ratio of 60:1. NH_4_Cl can hydrolyze in water to release H^+^, thereby lowering the solution pH [22]. As the liquid-to-solid mass ratio increased, the amount of NH_4_Cl solution available per unit mass of PG increased, which provided more favorable conditions for the dissolution of CaSO_4_·2H_2_O. In addition, the increased solution volume reduced the accumulation of dissolved Ca^2+^ and SO_4_^2−^ near the solid surface, thereby promoting mass transfer and facilitating further dissolution of the gypsum phase. These effects contributed to the increase in the Ca^2+^ leaching rate.

At 25 °C, with a liquid-to-solid mass ratio of 60:1 and a reaction time of 60 min, the Ca^2+^ leaching rate first increased significantly and then decreased slightly with increasing NH_4_Cl concentration. The maximum leaching rate of 81.25% was achieved at an NH_4_Cl concentration of 1.5 mol/L. With increasing NH_4_Cl concentration, the ionic strength of the leaching solution increased, and ion interactions such as Ca^2+^–Cl^−^ association became more pronounced. These effects can reduce the effective concentration of free Ca^2+^ in solution and shift the dissolution equilibrium of CaSO_4_·2H_2_O toward further dissolution, thereby increasing the Ca^2+^ leaching rate. However, when the NH_4_Cl concentration was further increased to 2.0 mol/L, the excessive ion concentration increased the solution viscosity [22], which hindered the diffusion of Ca^2+^ and SO_4_^2−^ and led to a slight decrease in the Ca^2+^ leaching rate.

At a liquid-to-solid mass ratio of 60:1 and an NH_4_Cl concentration of 1.5 mol/L, the Ca^2+^ leaching rate decreased with increasing reaction temperature after 60 min of leaching. The maximum leaching rate of 81.25% was obtained at 25 °C. This decreasing trend may be attributed to the reduced solubility of PG at elevated temperatures [23], which lowers the concentration of dissolved Ca^2+^ in the leaching solution and consequently decreases the Ca^2+^ leaching rate.

At 25 °C, with an NH_4_Cl concentration of 1.5 mol/L and a liquid-to-solid mass ratio of 60:1, the Ca^2+^ leaching rate initially increased and then tended to stabilize with increasing reaction time. The maximum leaching rate of 81.25% was achieved at 60 min. Sufficient reaction time is required for NH_4_Cl diffusion and its interaction with PG particles. Therefore, extending the leaching time promotes more complete calcium extraction and increases the Ca^2+^ leaching rate. However, excessive leaching time may lead to local supersaturation of Ca^2+^ and SO_4_^2−^, thereby promoting the reprecipitation of CaSO_4_. In addition, trace Fe^3+^ in PG may gradually dissolve during prolonged leaching and interact with SO_4_^2−^ to form hydrated ferric sulfate species, such as Fe_2_(SO_4_)_3_·xH_2_O [24]. These processes may reduce the amount of dissolved calcium remaining in solution, resulting in a slight decrease in the Ca^2+^ leaching rate.

In summary, the optimal conditions for Ca^2+^ extraction from PG using NH_4_Cl leaching were determined to be a reaction temperature of 25 °C, an NH_4_Cl concentration of 1.5 mol/L, a liquid-to-solid mass ratio of 60:1, and a leaching time of 60 min.

#### 3.1.2. Conversion Rate of Ca^2+^ in the Leaching Solution

**Figure 4:**
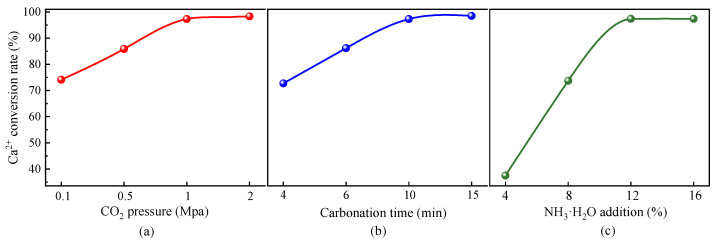
Effects of (**a**) CO_2_ pressure, (**b**) carbonation time, and (**c**) NH_3_·H_2_O addition on the Ca^2+^ conversion rate at 25 °C.

Figure 4 shows the Ca^2+^ conversion rate as a function of CO_2_ pressure, carbonation time, and NH_3_·H_2_O addition at 25 °C.

Under the conditions of 12 vol% NH_3_·H_2_O addition and a carbonation time of 10 min, the Ca^2+^ conversion rate increased markedly as the CO_2_ pressure increased from 0.1 to 1.0 MPa. However, when the pressure was further increased to 2.0 MPa, the additional improvement was limited, with the conversion rate reaching 98.34%. Compared with previous studies [12,13,14] using CO_2_ bubbling at atmospheric pressure, the present high-pressure carbonation process achieved a higher Ca^2+^ conversion rate within a shorter reaction time and under milder temperature conditions. In a sealed reactor, according to the ideal gas law, increasing the CO_2_ pressure at a constant temperature increases the amount of CO_2_ per unit volume. The higher CO_2_ concentration provides a stronger driving force for gas–liquid mass transfer [25], thereby promoting CO_2_ diffusion into the bulk liquid. In addition, according to Henry’s law, increasing the pressure enhances the solubility of CO_2_ in the solution. Dissolved CO_2_ reacts with water and undergoes stepwise dissociation to form carbonate species. Under alkaline conditions provided by NH_3_·H_2_O, the concentration of CO_3_^2−^ increases, which promotes its reaction with Ca^2+^ to form CaCO_3_ precipitates and thus increases the Ca^2+^ conversion rate. However, excessive CO_2_ dissolution at higher pressure may generate more carbonic acid and lower the system pH, shifting the carbonate equilibrium toward HCO_3_^−^. This may favor the formation of soluble calcium bicarbonate species and limit the further increase in the Ca^2+^ conversion rate.

Under the conditions of 12 vol% NH_3_·H_2_O addition and a CO_2_ pressure of 1.0 MPa, the Ca^2+^ conversion rate increased rapidly as the carbonation time was extended from 4 to 10 min. When the carbonation time was further extended to 15 min, the additional increase was limited, with the conversion rate ultimately reaching 98.51%. During the initial stage of carbonation, the Ca^2+^ concentration in the leachate was high, and the carbonate species generated from dissolved CO_2_ rapidly reacted with Ca^2+^ to form CaCO_3_ precipitates. At this stage, the reactant concentrations were relatively high, the diffusion distance was short, and the mass-transfer resistance was low, resulting in a rapid increase in the Ca^2+^ conversion rate. As carbonation proceeded, Ca^2+^ in the solution was progressively consumed, leading to a decrease in ion concentration and a reduced driving force for precipitation. Therefore, the increase in the Ca^2+^ conversion rate gradually slowed at longer carbonation times.

After carbonation for 10 min at a CO_2_ pressure of 1.0 MPa, the Ca^2+^ conversion rate initially increased substantially and then plateaued with increasing NH_3_·H_2_O addition. A Ca^2+^ conversion rate of 97.36% was achieved at 12 vol% NH_3_·H_2_O addition. As the NH_3_·H_2_O concentration increased, dissolved CO_2_ reacted with NH_3_·H_2_O to form ammonium carbonate species, which subsequently dissociated to provide CO_3_^2−^. Meanwhile, the increased NH_3_·H_2_O addition maintained an alkaline environment, promoting the conversion of dissolved CO_2_ into carbonate species. The resulting increase in CO_3_^2−^ concentration allowed more Ca^2+^ to precipitate as CaCO_3_, thereby increasing the Ca^2+^ conversion rate. However, at 16 vol% NH_3_·H_2_O addition, most of the Ca^2+^ had already precipitated as CaCO_3_, and the formed CaCO_3_ particles increased the mass-transfer resistance for the remaining unreacted Ca^2+^ and CO_3_^2−^. Consequently, the Ca^2+^ conversion rate showed little further increase. Therefore, 12 vol% NH_3_·H_2_O addition was selected as the optimal dosage because it enabled efficient Ca^2+^ conversion while avoiding reagent waste and the undesirable particle-size increase associated with excessive NH_3_·H_2_O addition [26].

In summary, a Ca^2+^ conversion rate of 97.36% was achieved under the optimized carbonation conditions: 12 vol% NH_3_·H_2_O addition, a carbonation temperature of 25 °C, a CO_2_ pressure of 1.0 MPa, and a carbonation time of 10 min.

#### 3.1.3. Crystal Morphology Under Optimal High-Pressure Carbonation Conditions

**Figure 5:**
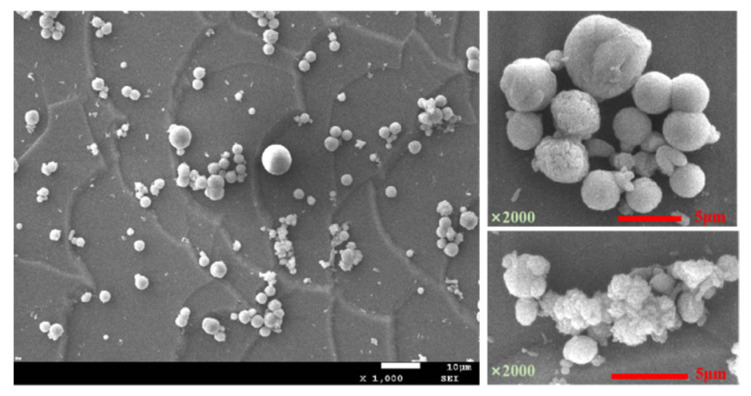
SEM images of CaCO_3_ under the optimized high-pressure carbonation conditions.

**Figure 6:**
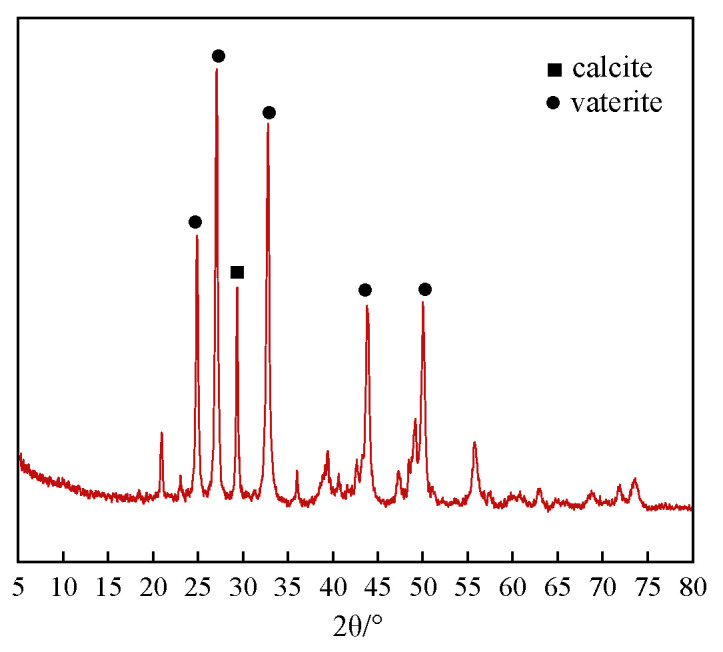
XRD pattern of CaCO_3_ under the optimized high-pressure carbonation conditions.

Figure 5 and Figure 6 show the SEM image and XRD pattern of calcium carbonate prepared from PG under the optimized high-pressure carbonation conditions, respectively. The obtained CaCO_3_ crystals were predominantly spherical, and band-like aggregates formed by the assembly of spherical particles were also observed. The XRD results indicated that the product was mainly composed of vaterite, with only trace amounts of calcite. In addition, the product contained a relatively high proportion of large particles, whereas fine particles were less developed.

In the aqueous carbonation system, NH_4_^+^ may interact with the hydrated surfaces of CaCO_3_ crystallites, thereby affecting particle aggregation and crystal growth. The adsorption of NH_4_^+^ may alter the surface interactions among CaCO_3_ particles, leading to crystal agglomeration, and may also modify the growth rates of specific crystal faces. These effects can change the crystal growth direction and contribute to the formation of the branched structures observed in the product [22]. In addition, NH_4_^+^ may adsorb onto active sites on calcite surfaces, such as the {104} facet, thereby blocking growth sites and inhibiting calcite development. According to the Ostwald ripening process, metastable vaterite can gradually transform into thermodynamically stable calcite with prolonged reaction time [5]. This transformation may partially offset the inhibitory effect of NH_4_^+^ on calcite growth, which explains the presence of weak calcite diffraction peaks in the XRD pattern.

### 3.2. Modulation of CaCO_3_ Polymorphs via Organic Additives

The CaCO_3_ crystals prepared under the optimized high-pressure carbonation conditions were predominantly vaterite; however, particle agglomeration and a non-uniform size distribution were observed. As a metastable CaCO_3_ polymorph with relatively low thermodynamic stability and crystallinity [5,6], vaterite is prone to phase transformation during storage or application, which may lead to performance degradation. Therefore, organic additives were introduced before carbonation of the Ca^2+^-rich leachate to regulate CaCO_3_ crystallization and improve the morphological uniformity of the products. In this study, the effects of these additives on the Ca^2+^ conversion rate, polymorph composition, and crystal morphology of the resulting CaCO_3_ were investigated.

#### 3.2.1. Ca^2+^ Conversion Rate in Leachate with Different Organic Additives

**Figure 7:**
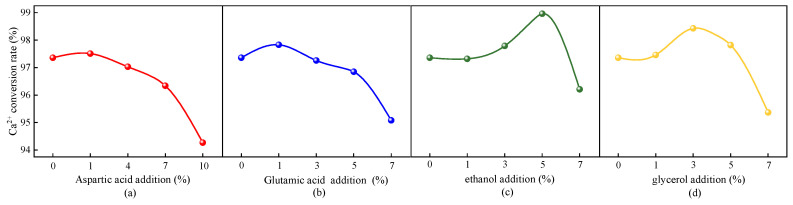
Effects of different organic additives on the Ca^2+^ conversion rate: (**a**) aspartic acid, (**b**) glutamic acid, (**c**) ethanol, and (**d**) glycerol.

Figure 7 shows the effect of additive concentration on the Ca^2+^ conversion rate.

With increasing aspartic acid and glutamic acid concentrations, the Ca^2+^ conversion rate initially increased slightly and then decreased. The maximum conversion rates of 97.51% and 97.83% were achieved at 1 wt% aspartic acid and 1 wt% glutamic acid, respectively. Aspartic acid and glutamic acid can provide nucleation sites for CaCO_3_ formation [27]. Therefore, at low concentrations, these additives promote CaCO_3_ nucleation and slightly enhance the Ca^2+^ conversion rate. However, when their concentrations exceed a certain threshold, steric hindrance may occur [27], which hinders the diffusion of Ca^2+^ and CO_3_^2−^ toward the growing crystal surfaces. In addition, aspartic acid and glutamic acid can form stable complexes with Ca^2+^ [20,27,28], thereby reducing the concentration of free Ca^2+^ in solution. According to the solubility product relationship of CaCO_3_, Ksp = [Ca^2+^][CO_3_^2−^], a decrease in free Ca^2+^ requires a higher CO_3_^2−^ concentration to initiate precipitation. This increases the precipitation barrier, inhibits CaCO_3_ formation, and ultimately leads to a decrease in the Ca^2+^ conversion rate.

The Ca^2+^ conversion rate also showed an initial increase followed by a decrease with increasing ethanol and glycerol concentrations. The maximum conversion rates of 98.96% and 98.43% were obtained at 5% ethanol and 3% glycerol, respectively. At low additive concentrations, both alcohols can increase solution supersaturation and reduce the nucleation barrier, thereby accelerating the early-stage nucleation of CaCO_3_ [29,30,31]. This promotes the reaction between Ca^2+^ and CO_3_^2−^, resulting in a slight increase in the Ca^2+^ conversion rate. However, as the alcohol concentration further increases, excessive alcohols significantly increase the solution viscosity [16,18]. The increased viscosity reduces the diffusion coefficients of Ca^2+^ and CO_3_^2−^ and slows their transport in the solution. Consequently, these ions cannot diffuse to the crystal growth sites in time, which retards the growth of CaCO_3_ nuclei. The abundant nuclei formed at the initial stage cannot obtain sufficient reactants for continuous growth, eventually leading to a decrease in the Ca^2+^ conversion rate.

#### 3.2.2. Crystal Morphology of CaCO_3_ with Different Organic Additives

**Figure 8:**
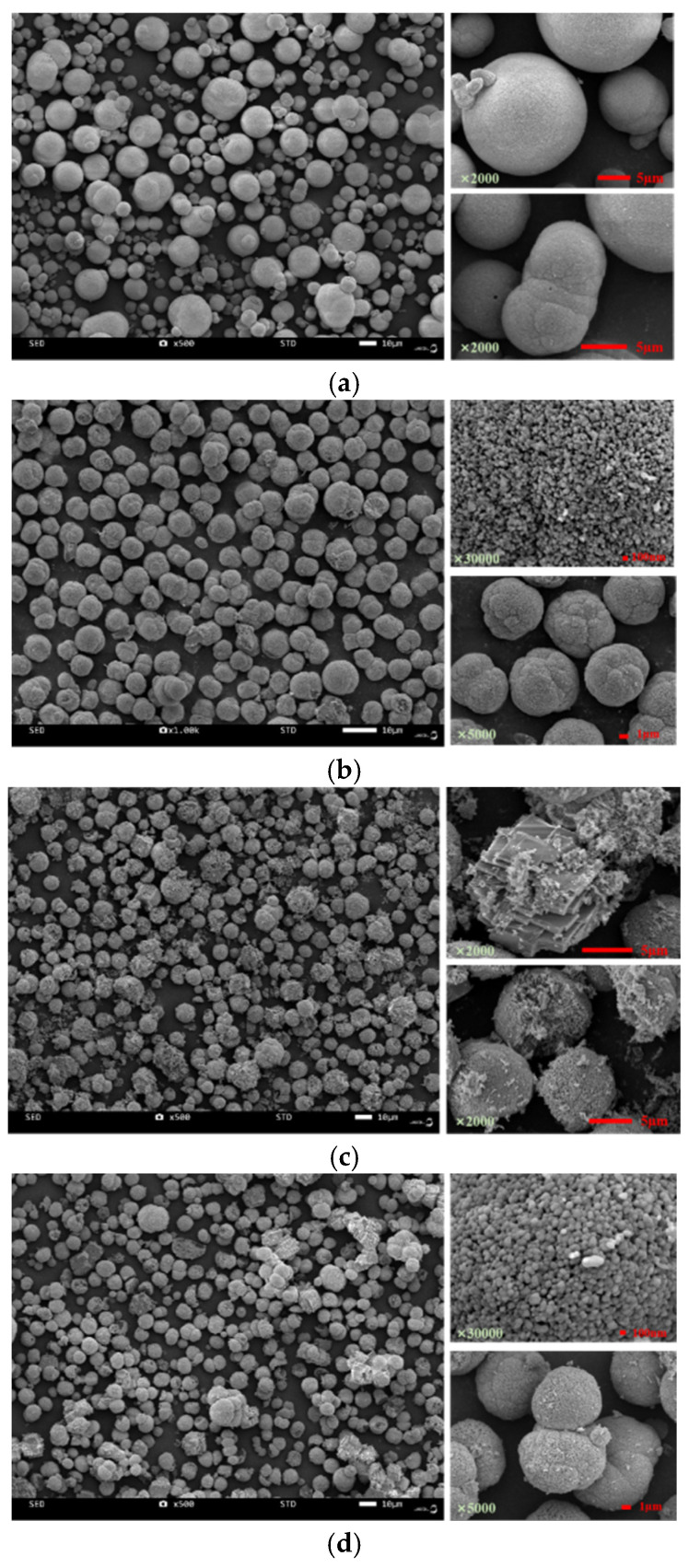
SEM images of CaCO_3_ synthesized with different organic additives: (**a**) 1 vol% aspartic acid, (**b**) 1 vol% glutamic acid, (**c**) 5 vol% ethanol, and (**d**) 3 vol% glycerol.

**Figure 9:**
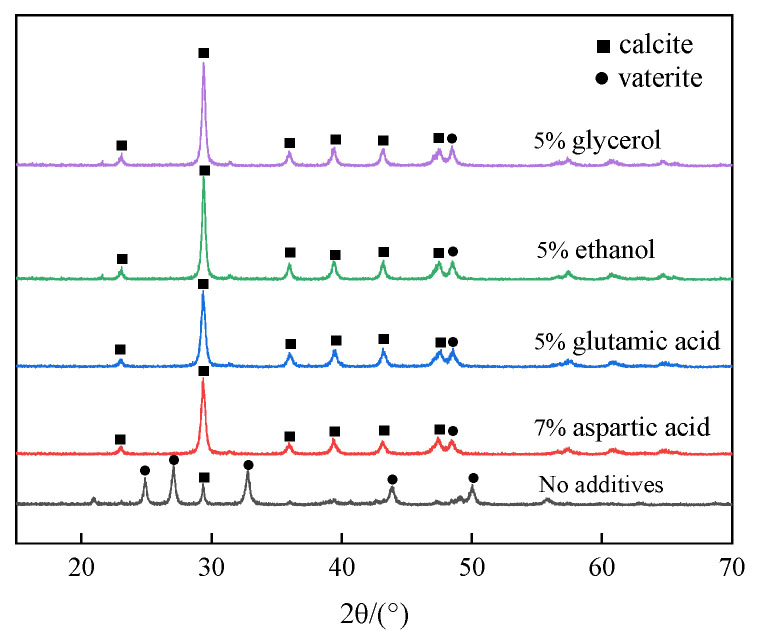
XRD patterns of calcium carbonate with different organic additives.

Figure 8 and Figure 9 show the SEM images and XRD patterns of CaCO_3_ prepared in the presence of different organic additives, respectively. With the addition of aspartic acid, glutamic acid, ethanol, or glycerol, calcite became the dominant polymorph, accompanied by trace amounts of vaterite. Previous studies [16,17] also reported that the addition of small amounts of ethanol or glycerol as organic additives can facilitate calcite formation during CaCO_3_ synthesis. The morphologies of the CaCO_3_ crystals varied noticeably under different additive conditions. Compared with the additive-free sample, the crystals prepared with organic additives were more fully developed, and their particle sizes were slightly reduced. In the presence of aspartic acid, the crystals were mainly spherical, with a small fraction of dumbbell-shaped particles. With glutamic acid, the crystals were predominantly quasi-spherical. In the ethanol-containing system, the crystals consisted of spherical particles, fine loose aggregates, and a small amount of cubic particles. In the glycerol-containing system, the crystals were mainly spherical.

After dissociation in solution, the negatively charged carboxylate groups of aspartic acid and glutamic acid can bind with Ca^2+^, thereby providing active sites for CaCO_3_ crystallization [32]. This interaction may lower the nucleation barrier of metastable vaterite and promote its formation. Owing to its crystal structure, vaterite tends to grow relatively uniformly in different directions, which favors the formation of spherical particles. In addition, amino acid molecules adsorbed on the surfaces of CaCO_3_ crystallites may impart a negative surface charge, enhancing electrostatic repulsion and weakening van der Waals attraction between particles [33]. As a result, well-dispersed spherical or quasi-spherical CaCO_3_ particles were obtained. Aspartic acid and glutamic acid can also selectively adsorb onto specific facets of thermodynamically stable calcite [20,21], thereby inhibiting the preferential growth of calcite. This may explain the presence of weak vaterite peaks in the XRD patterns. However, the carbonation process was followed by continuous stirring of the slurry for 2 h before vacuum filtration, during which dissolution and recrystallization may occur [34]. As aspartic acid and glutamic acid complex with dissolved Ca^2+^, the local ionic activity product may decrease, which can facilitate the transformation of metastable vaterite into thermodynamically stable calcite. This explains why calcite became the dominant phase in the final products.

Ethanol and glycerol can reduce the surface tension of the solution, and their hydroxyl groups may interact with or adsorb onto CaCO_3_ crystal surfaces. These effects can inhibit anisotropic growth and promote the simultaneous development of multiple crystal faces, resulting in smaller particles and the formation of fine, loose CaCO_3_ aggregates on the surfaces of spherical particles. Moreover, the addition of ethanol and glycerol increases the solution viscosity, which reduces interparticle collisions and slows Ostwald ripening [16,18]. This further suppresses excessive crystal growth and agglomeration, thereby improving the dispersion of CaCO_3_ particles. In addition, the hydroxyl groups of ethanol and glycerol may preferentially adsorb onto calcite facets [35,36]. This can stabilize calcite nuclei and inhibit vaterite growth, explaining the dominant calcite diffraction peaks observed in the XRD patterns.

In summary, the addition of all four organic additives led to a slight decrease in particle size, more complete crystal development, and a polymorphic transformation from vaterite to calcite. Among the additives tested, glutamic acid and glycerol showed stronger effects on crystal morphology regulation.

## 4. Conclusions

(1) The optimized process for preparing CaCO_3_ from PG by high-pressure carbonation was as follows. PG was first leached for 60 min at 25 °C using 1.5 mol/L NH_4_Cl with a liquid-to-solid mass ratio of 60:1, achieving a Ca^2+^ leaching rate of 81.25%. The resulting Ca^2+^-rich leachate was then carbonated for 10 min at 25 °C, a CO_2_ pressure of 1.0 MPa, and 12 vol% NH_3_·H_2_O addition, resulting in a Ca^2+^ conversion rate of 97.36%. The obtained CaCO_3_ crystals were predominantly vaterite with a quasi-spherical morphology, although particle agglomeration was observed.
(2) High-pressure carbonation was further performed using Ca^2+^-rich leachates containing organic additives, including aspartic acid, glutamic acid, ethanol, and glycerol. In all additive systems, the Ca^2+^ conversion rate initially increased and then decreased with increasing additive concentration. The optimal dosages were 1 wt% for both aspartic acid and glutamic acid, 5 vol% for ethanol, and 3 vol% for glycerol. The resulting CaCO_3_ products were predominantly calcite with quasi-spherical morphologies. The particles exhibited relatively uniform sizes and good dispersion. Among the tested additives, glutamic acid and glycerol showed stronger effects on crystal morphology regulation.

## Figures and Tables

**Table 1:** Chemical composition of raw PG (wt%).

SO_3_	CaO	SiO_2_	A1_2_O_3_	Fe_2_O_3_	K_2_O	CuO	P_2_O_5_	F	Loss
50.22	35.31	10.94	0.57	0.68	0.56	0.02	0.59	0.87	0.24

## Data Availability

The original contributions presented in this study are included in the article. Further inquiries can be directed to the corresponding author.
